# Hepatitis B in Pediatric Population: Observational Retrospective Study in Romania

**DOI:** 10.3390/life14030348

**Published:** 2024-03-07

**Authors:** Daniela Păcurar, Alexandru Dinulescu, Gheorghiță Jugulete, Alexandru-Sorin Păsărică, Irina Dijmărescu

**Affiliations:** 1 Department of Pediatrics, “Carol Davila” University of Medicine and Pharmacy, 020021 Bucharest, Romania; daniela.pacurar@umfcd.ro (D.P.); irina.dijmarescu@umfcd.ro (I.D.); 2 Department of Pediatrics, “Grigore Alexandrescu” Emergency Children’s Hospital, 011743 Bucharest, Romania; alexandru-sorin.pasarica@rez.umfcd.ro; 3 Department of Infectious Diseases 3, “Carol Davila” University of Medicine and Pharmacy, 020021 Bucharest, Romania; gheorghita.jugulete@umfcd.ro; 4 National Institute for Infectious Diseases “Prof. Dr. Matei Balș”, 021105 Bucharest, Romania

**Keywords:** hepatitis B, HBV, HCV, HDV, transaminases, ALT, AST, viraemia

## Abstract

Hepatitis B virus (HBV) is a frequent cause of chronic hepatitis worldwide, with an estimated 5.6 million children under 5 years being infected. In Romania, there are no available epidemiology reports on large cohorts in children. We aimed to assess the profile of pediatric chronic HBV infection in southern Romania. We conducted an observational retrospective study on 506 HBV-infected children. Based on alaninaminotransferase (ALT), HBV serology and viremia, we identified four states of the disease. We correlated age, gender, household HBV infection, coinfection with other viruses and laboratory parameters. Most patients were in a positive HBV envelope antigen (HBeAg) immune-active state (65.4%). Age at diagnosis was significantly lower for those with household infection (*p* < 0.05). ALT values were not significantly different between positive or negative HBeAg patients in the immune-active state (*p* = 0.780). ALT values were higher in patients with hepatitis D virus (HDV)-associated infection (*p* < 0.001). Children with a household HBV infection had a high viraemia more frequently when compared to those with no infected relative (79.3% vs. 67.4%) (*p* < 0.001), but the ALT values were not significantly different (*p* = 0.21). Most of the patients are in an immune-active state (high ALT, high viremia). The percentages of HBV- and HDV-associated infections are high, but lower than the reported prevalence in Romania in the general population.

## 1. Introduction

Pediatric hepatitis etiology is extensive and includes infections, autoimmune diseases, metabolic diseases, drugs or other etiologies. Hepatitis B virus (HBV) is a frequent cause of chronic hepatitis worldwide, resulting in an important public health issue through high morbidity and mortality, as well as its economic burden. The natural history of chronic HBV infection consists of four phases, defined by HBV surface antigen (HBsAg) and HBV envelope antigen (HBeAg) status, serum HBV deoxyribonucleic acid (DNA) and alanin-aminotransferase (ALT) levels. These phases are not necessarily sequential, nor mandatory for all patients; sometimes, a reversion to an earlier phase is noted. Host and viral factors; coinfection with other viruses such as hepatitis C virus (HCV), hepatitis D virus (HDV) or human immunodeficiency virus (HIV); family history; and other comorbidities, including obesity, can affect the natural course of HBV infection [1,2,3,4,5,6,7,8,9,10,11,12]. Another important thing to mention is that the covalently closed circular HBV-DNA (cccDNA) persists indefinitely in hepatocytes, and low-level viral replication or re-activation in some circumstances is possible; at the same time, the HBV genome may integrate in the host genome, generating risk for hepatocarcinoma (HCC) [13,14,15]. Routes of transmission include vertical (mother to child), early-life horizontal transmission (by bites, lesions and traditional procedures) and adult horizontal transmission (by sexual contact, intravenous drug use and medical procedure exposure) and are reported to varying degrees in every country [16,17,18]. Younger age at time of infection continues to be the most important predictor of chronic carriage. High infection (17%) and chronicity (54%) rates were reported in newborns of HBeAg-positive mothers despite concomitant active and passive immunization at birth; it was reported that the prevalence of HBeAg in asymptomatic carriers decreases with age, from around 90% in those younger than 15 years to 10% in patients over 40 years old [13,16,19].

According to the World Health Organization (WHO) estimates from 2022, one-third of the world’s population has been infected with HBV, and 257.5 (216.6–316.4) million people are chronic carriers of HBsAg, corresponding to a prevalence of 3.2%, of which only 14% were diagnosed, and only 8% of those eligible were treated. The 3.2% prevalence in adults is higher than in children (0.7% under 5 years old) due to vaccination [20]. The prevalence of chronic HBV infection varies widely, ranging from over 10% in some Asian and Western Pacific countries to under 0.5% in the United States and Northern European countries. Among European countries, Turkey (8%) and Romania (6%) have intermediate to high HBsAg carrier rates, followed by Bulgaria (4%), Latvia (2%) and Greece (2%). In the Slovak Republic, Poland, Czech Republic, Belgium, Lithuania, Italy and Germany, the HBsAg prevalence is reported to be 0.5–1.5%, and in the Netherlands, Estonia, Hungary, Slovenia and Norway, it is below 0.5% [16,21,22,23,24,25]. The WHO reports that most chronic HBV infections occur perinatally or in early childhood. They recommend timely birth dose (TBD) vaccination in newborns, within 24 h. HBV infection in children can be, at least in theory, efficiently prevented by vaccination, with the WHO reporting that after the introduction of the vaccine, the prevalence of HBV infection dropped from 5% to less than 1% in children under 5 years old. Another prophylactic measure they recommend is early specific immunoglobulin administration in newborns from HBV-infected mothers [25,26]. The most recent data estimate that approximately 85% of children under 1 year-old have been vaccinated with three doses for HBV, but only 46% received a TBD [20]. Even though in theory this prevention approach is efficient, there are studies that describe regions in Africa where TBD vaccination is not enough, urging for peripartum antiviral prophylaxis [27,28,29,30]. Since 2020, Tenofovir prophylaxis has been added to the list of recommendations for pregnant HBsAg-positive women with high viraemia, starting from 28 week of gestation until at least after birth. Even so, it is estimated that this approach is successfully applied in only 3% of cases [20,26,27,28,29,30].

To date, there are few epidemiological studies in Romania reporting the incidence, prevalence or characteristics of HBV infection both in adults and children. One study describes the change in incidence of HBV infection in Romania from the late 1980s until 2005. A dramatic decrease in HBV hepatitis incidence was identified. Overall, the incidence has decreased from 43 per 100,000 in 1989 to 8.5 per 100,000 in 2004; this decline is most prominent in children under 15, decreasing from 81 to 11 per 100,000 population a year during that period [31]. The last report regarding the prevalence of the hepatic viruses in Romania was issued in 2013, and the prevalence of HBV infection was estimated around 4.2%, an intermediate level compared to the 0.9% in the European Union and United Kingdom [32,33]. The most recent seroprevalence study for HBV infection in Romania was carried out in the period 2020–2022 by the National Institute of Public Health and the National Center for Surveillance and Control of Communicable Diseases in Romania, describing the presence of HBsAg as a marker of HBV infection in 6.2% of the adult population. The lowest prevalence of HBV infection was recorded in the 18–29 age group (2.3%) [34].

In Romania, vaccination for HBV is administered in four doses [35]. An old report, published in 2008, estimated that between 1995 (when routine HBV vaccination was introduced for newborns) and 2004, over 95% of children aged 0 to 18 years old were immunized [31]. The latest data available are from 2022, when the WHO and United Nations International Children’s Emergency Fund (UNICEF) estimated that approximately 97% of newborns have received TBD vaccination and 85% of children younger than 1 year old were immunized with all doses [36]. Prenatal screening for HBV is not routinely used in Romania. The prevalence of HBsAg-positive pregnant women in Romania was estimated by Popovici et al. at 5.1% in 2018 [37]. Approved treatment options for pediatric chronic hepatitis B according to the Romanian Health Ministry guide are as follows: Entecavir in children over 2 years old, Interferon alfa-2b after 3 years old and Tenofovir after 12 years old [38].

To date, in Romania, there are no epidemiology reports based on large cohorts of HBV patients providing data on age, routes of transmission, coinfections, comorbidities, family history of hepatitis viruses infection and socio-economic characteristics of pediatric patients with chronic viral hepatitis. We aimed to assess the clinical, biochemical and virological aspects of pediatric chronic HBV infection at the time of diagnosis and to outline the characteristics of the infected population. Also, we focused on classifying patients according to the phase of infection they were in, based on the status of HBeAg, viraemia and liver enzymes, to paint a bigger picture regarding the specific features of pediatric patients with chronic hepatitis B in southern Romania. The importance of this study is given by the fact that it is the first epidemiological study performed in Romania that covers pediatric chronic infection with HBV over a long period of time (30 years) including a large number of subjects.

## 2. Materials and Methods

We conducted an observational retrospective study which included pediatric patients aged 0 to 17 years old with HBV infection who were evaluated for the first time in the Pediatrics Department of “Grigore Alexandrescu” Emergency Children’s Hospital in Bucharest, Romania, between 1982 and 2023. The children included in this study were selected from those admitted to the hospital for persistently elevated transaminases, identified due to a known infection of a family member, accidentally when being evaluated for other reasons or referred to our department for further evaluation and treatment after being diagnosed in other centers. All patients were followed-up with for at least 6 months after the moment of their first positive AgHBs test.

We collected data on patients’ age, gender, area of residence, route of infection, HBV status of the mother and close relatives, risk factors for horizontal transmission (history of surgical or endoscopic procedures, blood transfusions, etc.) and laboratory tests—white blood cell count (WBC), liver enzymes, HBV serology (HBsAg, anti-HBs antibodies, HBeAg, anti-HBe antibodies), HBV-DNA—together with HCV, HDV and HIV status. To establish HCV, HDV and HIV status, antibodies were used for screening in all HBV-positive patients, and when found positive, the results were confirmed by RT-PCR.

Because the data have been collected over a 30-year time frame, the laboratory results were inhomogeneous, especially HBV-DNA data, which have been evaluated through different methods, providing results expressed in diverse units of measurement (through in situ hybridization, the viral load is quantified in pg/mL; for conversion to copies/mL, we used the formula 1 pg/mL = 2.86 × 10^5^ copies/mL; for conversion from copies/mL to IU/mL, we used the formula 1 IU = 5 copies) [39].

We analyzed whether the levels of transaminases, ALT and aspartate aminotransferase (AST) were correlated to the viral load for both HBV infection alone or in association with other viruses.

The patients were divided in four groups based on HBV serology, HBV viral load and ALT serum levels, according to the European association for the Study of the Liver (EASL) and ESPGHAN (Table 1).

The inclusion criteria were as follows: children (0–17 years old) with chronic HBV infection, defined as the persistence of HBsAg for more than 6 months during follow-up.

**Figure 1:**
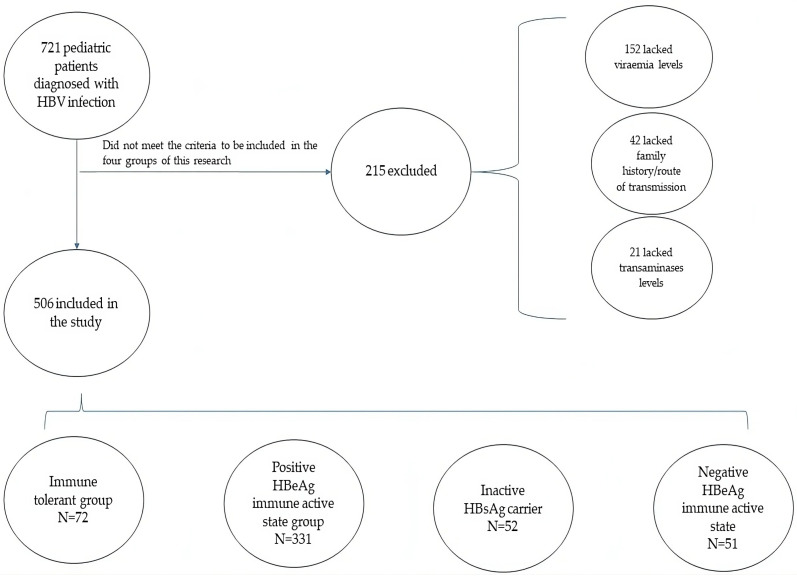
Selection of the subjects for the study.

The patients with incomplete data and who did not meet all the criteria to be included in one of the four groups of this research were excluded (Figure 1).

Vertical transmission was considered when mothers were HBsAg-positive and/or HBV infection was identified in other siblings, in the absence of medical interventions such as surgery or blood transfusion.

The groups were analyzed for correlations among age, gender, HBV infection of the mother or a close relative, coinfection with HCV/HDV and laboratory parameters.

All data were analyzed using IBM SPSS Statistics 25 and illustrated using Microsoft Office Excel/Word 2013. Quantitative variables were tested for normal distribution using the Kolmogorov–Smirnov test and were written as medians with interquartile ranges (IQRs). Quantitative variables were tested between independent groups using Mann–Whitney U tests. The Kruskal–Wallis test was used to determine significant differences between two or more groups of an independent variable. Fisher’s exact test was used to determine the nonrandom associations between categorical variables, with the Bonferroni method used for correction.

## 3. Results

### 3.1. Study Group Characteristics

In the Pediatrics Department of “Grigore Alexandrescu” Emergency Children’s Hospital in Bucharest, 721 pediatric patients aged 0 to 17 years old were evaluated for HBV infection from 1992 to 2023. Epidemiological data and laboratory test results were collected for all patients, but only 506 children met all the criteria to be included in the four groups of this research.

**Figure 2:**
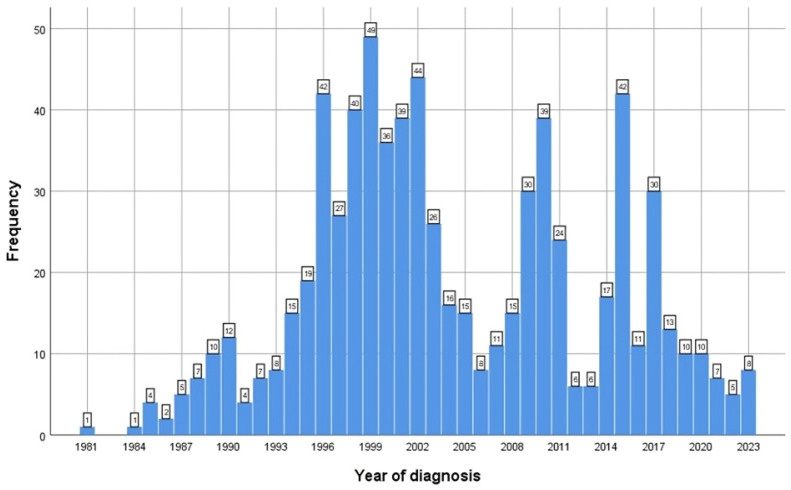
Number of children with chronic HBV infection by year of inclusion in the study.

Figure 2 presents the number of children diagnosed per year. Peaks can be identified from 1996 to 2002, 2009 to 2011 and 2014 to 2017.

For all 721 patients, when looking at the decades, most of them were diagnosed between 1992 and 2011 and between 2002 and 2011, and fewer were diagnosed after 2012.

**Figure 3:**
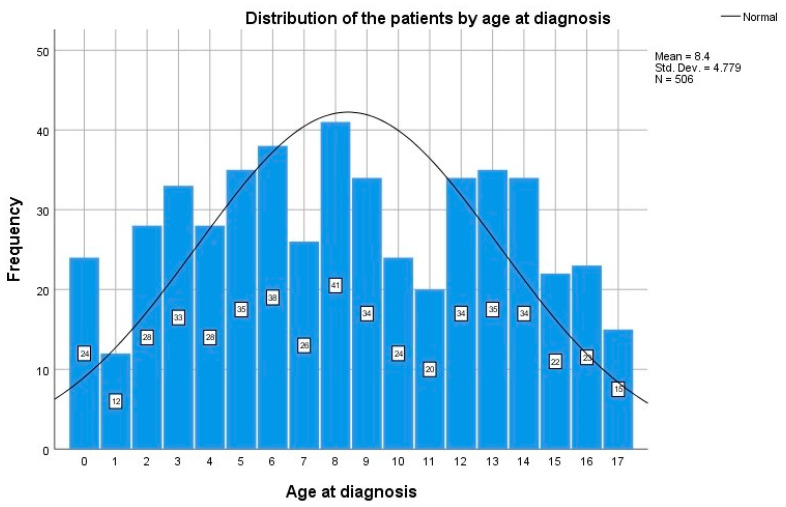
Distribution of children with chronic HBV infection by age group.

A total of 506 patients for whom all data were available at the first presentation (epidemiology, family history, biochemical and virologic laboratory tests) were identified. Out of the 506 selected patients, 301 (59.5%) were male and 205 (40.5%) were female. The age ranged from 0 to 17 years, with a median of 8 years (5–13) (Figure 3).

Out of 506 patients with HBV infection, 57 (11.2%) had an associated HDV infection, 5 (1%) of them had an associated HCV infection and 1 child (0.2%) had all three viruses (HBV, HDV and HCV).

### 3.2. Age at Diagnosis Analysis

Most children were diagnosed at age 8, followed by 6, 13 and 15 years old, but the distribution of the patients by age range was almost homogenous. There was no statistically significant difference for age at diagnosis according to gender (*p* = 0.332). Children were grouped by area of residence. Out of 506 children, 405 (80%) were residing in urban areas, while 101 (20%) originated from rural areas. Age at diagnosis was not significantly different between the two groups (*p* = 0.218).

**Figure 4:**
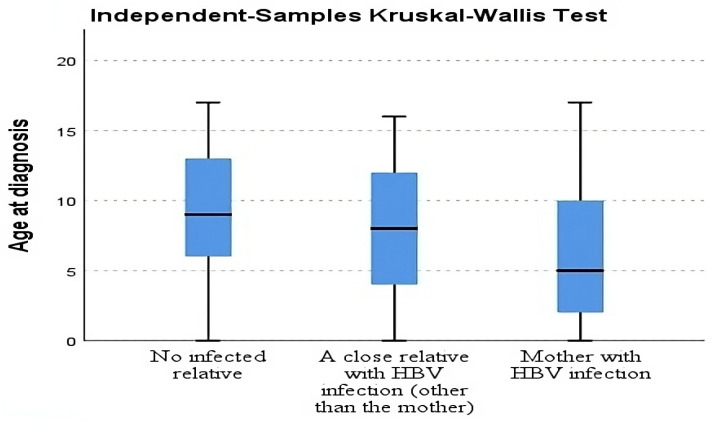
Family HBV infection status for chronic HBV-infected patients. The interior bars indicate the medians while the whiskers extend to the maximum and minimum of the data.

It was noted that 193 patients (38.1%) had at least one close relative with HBV infection, but this number also included children with infected mothers and vertical transmission. They were subsequently divided into two groups: 128 (66.3%) children with vertical transmission (HBsAg-positive mother) and 65 (33.7%) children with an infected close relative (other than the mother). We compiled a third group comprising 313 (61.9%) children with no household HBV contact. The age at diagnosis was significantly lower for those with vertical transmission (median age of 5 (2–10) years old) when compared to the other two groups (median age of 9 (6–13) years old for children without family history of HBV infection and 8 (4–12) years old for those with a close relative with HBV infection other than the mother) (*p* < 0.05). In the group including children who had a close relative with HBV infection (other than the mother), we identified a significantly lower age at diagnosis when compared to the group of children with no relative diagnosed with HBV infection (*p* = 0.023) (Figure 4).

### 3.3. Classification and Analysis by Type of Disease

Patients were divided into four groups, according to HBsAg and HBeAg status, viral load and ALT values. The characteristics of each group are presented in Table 2. In our cohort of 506 children, most children—331 (65.4%)—were in the positive HBeAg immune-active state. Fifty-two (10.3%) children were inactive HBsAg carriers were, and seventy-two (14.2%) were in an immune-tolerant state. The number of children with a negative HBeAg immune active-state was 51 (10.1%)—possibly pre-core mutant HBV infection (they were not evaluated for this).

The viral loads for each group according to stages of the disease were compared. The median viraemia of the negative HBeAg immune-active state group was significantly lower when compared to that of the positive HBeAg immune-active group and that of the immune-tolerant group (*p* < 0.001). There was no statistically significant difference regarding the serum viral load between the positive HBeAg immune-active group and the immune-tolerant one (*p* = 0.082).

There was no statistically significant difference for the age at diagnosis among disease state groups (*p* = 0.124).

The negative HBeAg immune-active state and the immune-tolerant state were less frequent among those with vertical transmission (9.8% and 34.7%) (*p* = 0.014).

### 3.4. Analysis of the ALT Levels

ALT values were elevated for 382 (75.5%) of the patients (Table 3). The values had a median of 76 (39.2–130.5) U/L. Most of them (48.2%) had mild–moderate cytolysis (ALT 1-3 UNL).

**Figure 5:**
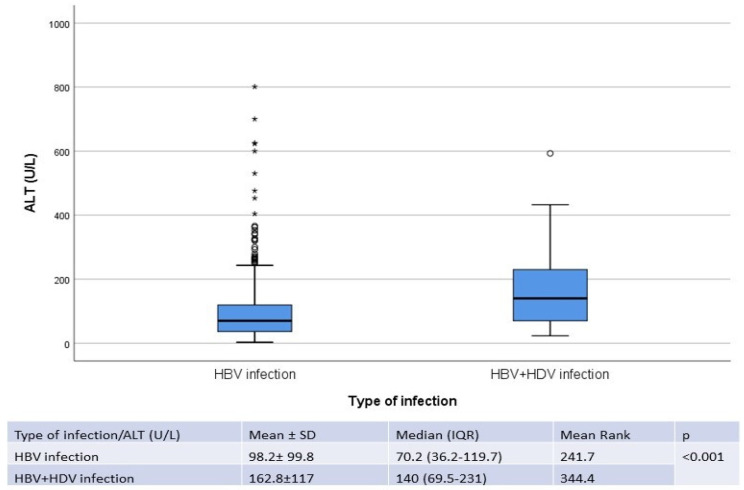
Value of ALT according to the absence/presence of HDV infection in children with chronic infection with HBV. The interior bars indicate the medians while the whiskers extend to the maximum and minimum of the data; ◦ = outlier; * = far outlier.

No correlation was identified for the value of ALT according to the age of patients (*p* = 0.722). The values of ALT were not significantly different between positive HBeAg patients and negative HBeAg ones in the immune-active state (*p* = 0.780). The values of ALT were higher in patients with HDV-associated infection (*p* < 0.001) (Figure 5).

The De Ritis ratio (AST/ALT) was higher in patients with chronic infection, either HBeAg-positive or -negative, with a median of 1 (1–1.2), respectively, 1.2 (0.9–1.5), when compared to those with chronic hepatitis, with a median of 0.7 (0.5–1), respectively, 0.8 (0.6–1) (*p* < 0.005).

### 3.5. Analysis of the Groups by Viraemia Level

The viraemia (measured in IU/mL) had a median of 2.8 × 10^8^ (9 × 10^4^–2.2 × 10^9^) IU/mL. Viral load was not significantly different when analyzed according to gender (*p* = 0.191). Based on the serum level of HBV-DNA, infected patients were further divided (Table 4), and for each group we analyzed the serum level of liver enzymes and type of infection (HBV alone or double infection with HBV and HDV). We report a viral load higher than 10^6^ IU/mL for 71.9% of patients. Viraemia was not significantly different according to age (*p* = 0.483).

The data in Table 4 show that patients with both HBV infection alone and HBV + HDV infection have high viral loads (>106 IU/mL). However, a higher percentage of patients with single infection (HBV) had viral loads > 10^6^ IU/mL when compared to the ones with double infection (HBV + HDV) (73.7% vs. 58.6%) (*p* = 0.023).

ALT levels were significantly lower for the group with HBV-DNA (<2 × 10^3^) when compared to the other groups with different viral loads; also in addition, ALT values were significantly lower for the group with HBV-DNA (2 × 10^3^–2 × 10^4^) when compared to the groups with higher viral loads (*p* < 0.05). There was no statistically significant difference among groups with a viral load higher than 2 × 10^4^ (*p* > 0.05). Children with an HBV-infected close relative had a viral load >10^6^ IU/mL more frequently compared to those with no HBV-infected relative (79.3% vs. 67.4%) (*p* < 0.001). ALT values were not significantly higher in children with household HBV contact (*p* = 0.21) when compared with those without close relatives with HBV infection (Table 5).

## 4. Discussion

The current research, which included 721 new cases of children with HBV infection, revealed an increase in diagnosed cases after the year 1990, with three peaks in number of cases/year—first between 1996 and 2002, second between 2009 and 2011 and third between 2014 and 2017, corresponding to national screening programs for hepatitis virus infection. Even though HBV vaccination was implemented in Romania in 1995 for all newborns and children and at-risk populations, there was no significant decrease in the number of diagnosed HBV cases in the first 10 years. A decline in new cases was noticed only after 20 years from the start of vaccination [40]. There are literature reports of a decreased diagnosis rate of HBV infections during the COVID-19 pandemic [41]. Our study covers only 2 years of that period, 2020 and 2021, with only a small decrease observed during these 2 years when compared to the 2018–2019 period (six vs. eight new cases/year). The current study is not a general population incidence study, and the high number of diagnosed HBV patients can be justified by the fact that most patients with liver cytolysis are referred to our department as a hepatology center. The mean age at diagnosis for our study group was 8 years and 3 months. In 36 patients, the diagnosis was established before the age of 2, the transmission being vertical (mother-to-child), regardless of the status of specific prophylaxis. Four of those thirty-six children (11.1%) were diagnosed before 1995, when no specific prophylaxis was available (HBV vaccination was started in 1995 in Romania), and twenty-one (58.3%) of them were confirmed before 2006 (the year when HBV-specific immunoglobulins were approved). Out of 506 children, 405 (80%) originated from urban areas and 101 (20%) from rural areas, the important difference potentially being the result of several factors such as a distinct level of medical education, implementation of screening programs for pregnant women or higher accessibility to medical services in urban areas.

There is a known gender disparity in the adult population with HBV infection, sex hormones being responsible for this. The immune clearance rate is considered to be higher in women, and male gender is an important risk factor for chronic infection, progression to cirrhosis and HCC. Trevisan et al. concluded that there is a sexual disparity in response to vaccination against HBV as well, especially for females vaccinated when older than 1 years old, who exhibited a stronger immune response [42]. Our research supports this idea, reporting 301 (59.5%) male patients and 205 (40.5%) female patients. Ruggier et al. reported a lower serum viral load in female carriers when compared to male ones, but we found no statistically significant difference between sex and viraemia levels (*p* = 0.191) [43,44,45,46].

An HBV-infected close relative is an important risk factor for infection in children and is labeled as the intra-familial spread of infection. A study conducted by Ucmak et al. analyzed intra-familial HBV infection rates in 2133 family members of infected subjects from Turkey and concluded that intra-familial horizontal spread is an important route of transmission. The authors found that the rate of HBsAg-positive infection was higher in children with positive mothers when compared to those with positive fathers (19.7% vs. 5.4%) and was even higher when both parents were infected (26.6%). In families with more infected members, the risk of HBV infection was reported to be even higher [47]. In our study group, 193 children (38.1%) had at least one close relative with HBV infection. Vertical transmission from infected mothers was reported in 128 patients (25.3%), and the other 65 (12.8%) had an infected close relative, with probable household transmission. In the group with an HBV-infected close relative other than the mother, age at diagnosis was lower than in the group with no infected relatives, probably because in both groups with infected family members, screening tests for children were performed earlier. No statistically significant difference was identified between the age at diagnosis and the gender of patients in our cohort (*p* = 0.332). We found no research that compared viral loads between the group of children with household contacts and the group of patients with no familial history of HBV infection. The viral load (IU/mL) was significantly higher in children who had a member of the household with HBV infection, when compared with those without familial contacts (~1.1 × 10^8^ (10^6^–5.3 × 10^8^) vs. ~1.4 × 10^7^ (3.4 × 10^5^–1.5 × 10^8^); *p* < 0.001). We found no statistically significant difference regarding ALT values between groups with or without household exposure to HBV (*p* = 0.21). It is now known that the younger the age at infection, the higher the risk of chronic and persistent HBV. Subsequently, longtime infection leads to several complications: HBV-DNA integration in the host cells, cirrhosis, HCC, specific immune reaction, etc. [48,49,50].

Chronic hepatitis D is the most severe viral hepatitis, with persistently high values of liver enzymes and indolent progression toward cirrhosis. Tseligka reviewed many studies in adults and children infected with HDV and noticed that the risk of cirrhosis in patients coinfected with HBV and HDV are two-fold higher when compared to the risk of patients with HBV mono-infection. Even patients with undetectable HDV-RNA have a risk of cirrhosis of 22% [51]. The prevalence of anti-HDV antibodies in people infected with HBV is very high in Romania, estimated at 23% in 2015 [52]. There are few studies on HDV’s prevalence in children, but because of the lower HBV prevalence in children when compared to adults, HDV prevalence is also lower. Out of 506 patients with HBV infection included in our study, 57 (11.2%) had an associated HDV infection, lower than the reported prevalence in Romania for the general population, but still a high prevalence for children. HBV-HCV coinfection is not uncommon, because the two viruses share the same mode of transmission. There are many studies that estimate the prevalence of this coinfection, with many discrepancies, ranging from 0.7% in Egypt to 16% in India. A study from Brazil states that HBV-HCV coinfection is present in 7–15% of patients with chronic HBV, while other studies from European and Asian countries estimate that 10–15% of patients with chronic HBV infection have an associated HCV infection [53,54]. In our research, five (1%) patients had HBV + HCV-associated infection. We found no data regarding the prevalence of triple infection (HBV, HCV, HDV) in the literature, but there are some reported cases, especially from Mongolia, a highly endemic country for all three viruses. Lorenc et al. has published two cases with triple infection in Poland [55,56]. In our study, one child (0.2%) had all three viruses. The values of ALT were significantly higher in those with HBV + HDV double infection when compared to those with HBV infection alone, with a median of 140 U/L (69.5–231) vs. 70.23 U/L (36.25–119.75) (*p* < 0.001).

The European Association for the Study of the Liver (EASL) in 2017 and ESPGHAN in 2020 classified infection with HBV into four types, as stated above [1,57]. Following this classification, the same criteria were applied in our research based on the HBeAg status, viral load and liver enzyme values. Most of the patients, 331 (65.4%), were in an immune-active phase, with positive HBeAg. Fifty-one (10.1%) patients were in the immune-active state with negative HBeAg. Patients in an immune-active state of the disease represent 75.4% (382 children) of the total and could be considered for specific medication. One-quarter of patients included in our survey were not eligible for treatment: 52 children (10.3%) were identified to be inactive HBV carriers or have an HBeAg-negative infection, while 72 patients (14.2%) were in an immune-tolerant state. For these categories, a close follow-up is mandatory due to many potential early complications. In young immune-tolerant individuals, a significant HBV-specific T cell response activity is demonstrated; thus, the idea that children infected at birth or in early childhood will remain in an immune-tolerant state with no disease or minimal disease for at least 10 years is considered to be improbable nowadays [58,59]. Even inactive carriers may be at risk for HCC because of HBV DNA integration in the host genome, which can occur in all stages of the disease, starting from the very first days of infection [60].

ALT is used as an indicator of liver injury. The high values of ALT in adult studies are associated with the development of cirrhosis, and the level of ALT is an important factor in the decision to start treatment [61,62,63]. In our research, 382 (75.5%) children had elevated values of ALT (considering normal values for age and sex), so they were eligible for treatment. Cytolysis was mild–moderate (1–3× ULN) in 48.2% of them. The EASL 2017 Clinical Practice Guidelines on the management of HBV infection recommend checking the values of ALT at specific intervals (6 or 12 months) based on the HBV-DNA levels, HBeAg status and HBsAg levels [1]. Kumar et al. analyzed 207 patients with acute or chronic liver disease and reported that 80% percent of those with positive HBeAg had high ALT [64]. ALT was not significantly different when comparing the group including inactive HbsAg carriers and the one including patients in an immune-tolerant state, nor when comparing the groups consisting of patients with immune-active disease, whether HBeAg-positive or HBeAg-negative (*p* > 0.005). A correlation between HBV-DNA and ALT values has been reported, with a reduction in the viral load in patients with low ALT values [65,66], a correlation similar to the one we report. Groups including children with lower viraemia (<2 × 10^3^ and 2 × 10^3^–2 × 10^4^) had lower ALT values than the groups with high viral load (>2 × 10^4^) (*p* < 0.05). We could consider that viral loads lower than 2 × 10^4^ UI/mL (or <10^5^ copies/mL) are associated with the absence of liver cytolysis and minimal hepatic lesions. However, screening is needed, and we recommend it be performed every 6 months to ensure an early administration of antiviral therapy when needed. The highest values for transaminases were identified in the group including patients with an HBV-DNA of 2 × 10^5^–1 × 10^6^, followed by the group with an HBV-DNA of 2 × 10^4^–2 × 10^5^.

The viral load is a predictive factor for the outcome and the mortality of HBV infection, with higher values of viraemia being associated with a higher probability of an unfavorable outcome, greater prevalence of HCC and a higher mortality rate [67,68,69,70]. In our study group, we report 434 patients (85.8%) with a viral load higher than 2 × 10^4^ IU/mL, out of which 346 (71.6%) had a viral load >10^6^ IU/mL. Data from the literature indicate that there is a statistically significant relationship between HBV viral load and mutations in the pre-core region [71,72]. We noticed that the median viraemia in children in an immune-active state with negative HBeAg was lower when compared to the group of patients in an immune-active state with positive HBeAg and the group of children in an immune-tolerant stage (*p* < 0.001). There was no statistically significant difference between patients with immune-tolerant disease and patients in an immune-active state with positive HBeAg hepatitis (*p* = 0.082). Studies that assess the viral load in patients with mono-infection (HBV) in comparison to the ones with double infection (HBV + HDV) report that patients with an associated HDV infection have a lower HBV viraemia [73]. This result is confirmed by our research, as we found that in patients with HBV infection alone, a viral load >10^6^ IU/mL was more prevalent than in those with an associated HDV infection (73.7% vs. 58.6%) (*p* = 0.023).

### Limitations

This study includes patients from an extended period of time, approximately 30 years, and thus some were lost during follow-up. Because of technological limitations, we did not explore the presence/absence of mutations in the pre-core region in our study group.

## 5. Conclusions

In Romania, there are no studies regarding the profile of pediatric patients with HBV infection. Most of the identified patients are in an immune-active state, with high transaminases and high viral loads, and they are eligible for specific treatment. A significant percent of HBV patients had an HDV-associated infection, lower than the reported prevalence in Romania for the general population, but still a high prevalence for children. Familial transmission remains high (vertical or horizontal from household contacts), thus nonspecific and specific prophylaxis methods should be promoted. Even after vaccination for HBV was started, the incidence of HBV-positive children did not decrease in the first 10 years. This study highlights the increased number of patients eligible for treatment in the southern part of Romania, being helpful in public health measures for prevention and cost estimation of pediatric HBV infection in this country.

## Figures and Tables

**Table 1:** HBV infection groups based on EASL and ESPHGHAN classification [3,8].

Group	HBsAg	HBeAg	HBV DNA	ALT Values
Immune tolerant (positive HBeAg chronic infection)	positive	positive	>20,000 IU/mL	Normal
Positive HBeAg immune-active state (positive HBeAg chronic hepatitis)	positive	positive	>2000 IU/mL	Elevated
Inactive HBsAg carrier (negative HBeAg chronic infection)	positive	negative	<2000 IU/mL	Normal
Negative HBeAg immune-active state (negative HBeAg chronic hepatitis)	positive	negative	>20,000 IU/mL	Elevated

HBV—Hepatitis B virus; HbsAg—Hepatitis B surface antigen; HBeAg—Hepatitis B envelope antigen; ALT—alanine aminotransferase; DNA—deoxyribonucleic acid.

**Table 2:** The classification of HBV infection by type of disease.

Marker	Immune-Tolerant	Positive HBeAg Immune-Active State	Inactive HBsAg Carrier	Negative HBeAg Immune-Active State
	Positive HBeAg Chronic Infection	Positive HBeAg Chronic Hepatitis	Negative HBeAg Chronic Infection	Negative HBeAg Chronic Hepatitis
HBsAg	+	+	+	+
HBeAg	+	+	-	-
Number of patients (%)	72 (14.2%)	331 (65.4%)	52 (10.3%)	51 (10.1%)
Mean (±SD) and median (IQR) age at diagnosis (years)	8.9 ± 4.8 9 (4–13)	8.1 ± 4.8 8 (4–12)	8.1 ± 4.7 8 (4.25–12)	9.6 ± 4.1 10 (6–13)
Mean (±SD) and median (IQR) ALT	N 27.3 ± 6.8 27.5 (23–32.4)	↑ 132.9 ± 111.8 92.4 (62–159)	N 23 ± 5.4 22 (20–25.7)	↑ 122.6 ± 72.6 103 (69–161)
De Ritis ratio (AST/ALT)	1.19 (0.91–1.53)	0.76 (0.57–1)	1 (1–1.28)	0.78 (0.61–0.99)
HBV DNA (IU/mL) Mean ± SD	~1.7 × 10^9^ ± 6.9 × 10^9^	~9 × 10^8^ ± 5.7 × 10^9^	negative	~8.6 × 10^7^ ± 3 × 10^8^
HBV DNA (IU/mL) Median (IQR)	~5.8 × 10^7^ (1 × 10^6^–5.1 × 10^8^)	~7.8 × 10^8^ (4.67 × 10^6^–2.5 × 10^8^)	negative	~1.1 × 10^6^ (3.2 × 10^5^–2 × 10^7^)
Vertical transmission	25	85	13	5
HBV + HDV	1	33	6	18

HBV—Hepatitis B virus; HDV—Hepatitis delta virus; HbsAg—Hepatitis B surface antigen; HBeAg—Hepatitis B envelope antigen; ALT—alanine aminotransferase; DNA—deoxyribonucleic acid; IQR—interquartile range; SD—standard deviation, N—normal values, ↑—elevated values.

**Table 3:** ALT values for patients with HBV infection.

ALT	Normal Values	1–1.5 × U/L	1.5–3 × U/L	3–5 × U/L	5–10 × U/L	>10 × U/L
N (%)	124 (24.5%)	93 (18.4%)	151 (29.8%)	74 (14.6%)	53 (10.5%)	11 (2.2%)

ALT—alanine aminotransferase.

**Table 4:** Distribution of the study group according to viraemia, liver enzyme serum level and HBV infection with/without associated HDV infection. The values marked in bold represent the proportions that differ significantly from each other, according to Fisher’s exact test.

HBV-DNA	<2 × 10^3^	2 × 10^3^–2 × 10^4^	2 × 10^4^–2 × 10^5^	2 × 10^5^–1 × 10^6^	>10^6^
Total HBV (No/%)	44 (8.7%)	28 (5.5%)	26 (5.1%)	44 (8.7%)	364 (71.9%)
HBV alone	38 (8.5%)	26 (5.8%)	21 (4.7%)	**33 (7.4%)**	**330 (73.7%)**
HBV + HDV	6 (10.3%)	2 (3.4%)	5 (8.6%)	**11 (19%)**	**34 (58.6%)**
ALT (U/L)	21.5 (20–25.7)	48.5 (24–104.7)	94.5 (51.5–256)	111.5 (58.2–152.5)	80 (49–133.7)

HBV—Hepatitis B virus; HDV—Hepatitis delta virus; ALT—alanine aminotransferase; DNA—deoxyribonucleic acid.

**Table 5:** Viraemia and serum liver enzymes in children with household HBV infection.

Group	N (%)	Mean Age ± SD	Median Age (IQR)	Median (IQR) of DNA-HBV (IU/mL)	Mean (±SD) and Median (IQR) of ALT
No infected relative	313 (61.9%)	9.3 ± 4.5	9 (6–13)	~1.4 × 10^7^ (3.4 × 10^5^–1.5 × 10^8^)	110.5 ± 97.9 80 (43.2–149)
Household HBV infection (total)	193 (38.1%)	6.8 ± 4.7	6 (3–10)	~1.1 × 10^8^ (10^6^–5.3 × 10^8^)	97.69 ± 112.6 66 (35.5–110.2)
A close relative with HBV infection (other than the mother)	65 (12.8%)	7.8 ± 4.3	8 (4–12)	~2.8 × 10^7^ (4.3 × 10^5^–2.2 × 10^8^)	98.9 ± 100.8 78 (42–120)
Mother-to-child transmission	128 (25.3%)	6.4 ± 4.8	5 (2–10)	~1.6 × 10^8^ (1.3 × 10^6^–7.2 × 10^8^)	97 ± 118.6 64 (32.9–105.7)

HBV—Hepatitis B virus; DNA—deoxyribonucleic acid; IQR—interquartile range; SD—standard deviation.

## Data Availability

The data can be shared up on request.
