# Correction: Păcurar et al. Hepatitis B in Pediatric Population: Observational Retrospective Study in Romania. *Life* 2024, *14*, 348

**DOI:** 10.3390/life14091075

**Published:** 2024-08-28

**Authors:** Daniela Păcurar, Alexandru Dinulescu, Gheorghiță Jugulete, Alexandru-Sorin Păsărică, Irina Dijmărescu

**Affiliations:** 1 Department of Pediatrics, “Carol Davila” University of Medicine and Pharmacy, 020021 Bucharest, Romania; daniela.pacurar@umfcd.ro (D.P.); irina.dijmarescu@umfcd.ro (I.D.); 2 Department of Pediatrics, “Grigore Alexandrescu” Emergency Children’s Hospital, 011743 Bucharest, Romania; alexandru-sorin.pasarica@rez.umfcd.ro; 3 Department of Infectious Diseases 3, “Carol Davila” University of Medicine and Pharmacy, 020021 Bucharest, Romania; gheorghita.jugulete@umfcd.ro; 4 National Institute for Infectious Diseases “Prof. Dr. Matei Balș”, 021105 Bucharest, Romania

**Text Correction**

There was an error in the original publication; the prevalence of HBV infection in Turkey was initially reported as 8% [1].

The following correction has been made to the Introduction section, paragraph 2:

The prevalence of chronic HBV infection varies widely, ranging from over 10% in some Asian and Western Pacific countries to under 0.5% in the United States and Northern European countries. Among the European countries, Romania (6%) has intermediate to high HBsAg carrier rates, followed by Turkey (~4.5%), Bulgaria (4%), Latvia (2%), and Greece (2%) [21,22].

The citations [2,3] have now been inserted, with this correction, the order of some references has been adjusted accordingly. The authors state that the scientific conclusions are unaffected. This correction was approved by the Academic Editor. The original publication has also been updated.
